# Shortened Telomere Length as a Risk Factor for Idiopathic Pulmonary Fibrosis: A Meta-Analysis

**DOI:** 10.2174/0118743064421488251017061020

**Published:** 2026-02-09

**Authors:** Fanny Fachrucha, Farhana Ibrahim Syuaib, Arini Purwono, Fariz Nurwidya, Sita Laksmi Andarini, Erlina Burhan, Wiwien Heru Wiyono

**Affiliations:** 1 Department of Pulmonology and Respiratory Medicine, Universitas Indonesia, Jakarta, Indonesia; 2 Department of Pulmonology and Respiratory Medicine, Faculty of Medicine, Universitas Indonesia, Jakarta, Indonesia

**Keywords:** Idiopathic pulmonary fibrosis, Interstitial lung disease, Telomere length, Shortened telomere

## Abstract

**Background:**

Idiopathic Pulmonary Fibrosis (IPF) is a progressive lung disease with limited life expectancy after diagnosis. The median survival time ranges from 2 to 4 years, indicating a poor prognosis. Multiple telomere-related genes that cause telomere shortening have been associated with a significant percentage of IPF cases. This review aims to analyze the association of short telomere length with IPF incidence.

**Methods:**

A systematic online search was conducted on PubMed, Scopus, and Cochrane. Articles that met the criteria were included. Quality of included literature was assessed using the Newcastle–Ottawa Scale (NOS). The pooled standard mean difference (SMD) with 95% confidence interval (CI) of telomere length was calculated using a random-effect model.

**Results:**

Six original studies containing 622 IPF patients and 544 controls were included in the meta-analysis. The study designs were case control and cohort. Pooled analysis showed shorter telomere length in IPF patients compared to controls (SMD: -0.84, 95%CI -1.21 to -0.48, Z = 4.55, *p* < 0.00001). Subgroup analysis showed that steeper telomere shortening was found in lung tissue compared to peripheral blood sample. The findings suggested that telomere length may be closely associated with the pathogenesis of pulmonary fibrosis.

**Discussion:**

Repeated cell divisions gradually shorten telomeres that lead to senescence and apoptosis. Premature senescence disrupts the balance of lung epithelial cells, potentially activating lung remodeling processes that result in fibrotic damage through senescence-associated secretory phenotype (SASP).

**Conclusion:**

This study shows significant shorter telomere lengths in IPF patients compared to healthy controls that suggest telomere as a risk factor for IPF occurrence. These findings highlight the value of telomere assessment not only for early detection but also as a potential predictive biomarker for clinical outcomes.

## 1 INTRODUCTION

Idiopathic Pulmonary Fibrosis (IPF) is a progressive lung disease with limited life expectancy after diagnosis. The disease is characterized by continuous scarring of the lung parenchyma, leading to a decline in pulmonary function [1, 2]. These injuries stimulate alveoli to release cytokines and growth factors that promote recruitment, proliferation, and differentiation of lung fibroblasts into myofibroblasts [3, 4]. This cascade creates a loop that leads to excessive collagen deposition [5], along with alveolar reepithelialization failure [6]. IPF is recognized as a rare disease [7]; however, its incidence has increased in recent years [8]. This rise is due to improvements in diagnostic methods and the aging of the population [1].

IPF is the most prevalent and morbid disease among Idiopathic Interstitial Pneumonias (IIPs) [9]. The median survival time ranges from 2 to 4 years, indicating a poor prognosis [10]. Pulmonary function can rapidly decline, leading to respiratory failure, whereas 10-15% of patients experience an unusually rapid decline within months [10]. Epidemiological studies in North American and European populations report 3 to 9 cases per 100,000 people per year [10]. The Incidence in Asia and South America is estimated to be lower, around 0.5 to 4.2 cases per 100,000 people per year [11]. However, these numbers continue to rise, mainly among elderly individuals over 65 years [12]. Additionally, male gender is also a prominent risk factor for IPF incidence [13].

Multiple telomere-related genes that cause telomere shortening have been associated with a significant percentage of IPF cases [14]. Mutations in telomere genes are found in 25% of familial cases and 1% to 3% of sporadic cases [15]. Moreover, shortened telomeres are also observed in sporadic cases without mutations [15]. It is reported that 10% of the patients have telomeres as short as those in mutation carriers. These findings lead to a poor prognosis due to impaired tissue repair [16]. Telomere shortening has also been observed in studies of other lung diseases with fibrosis phenotypes [17].

Given the fragmented evidence on telomere length and IPF, a meta-analysis is warranted to synthesize available findings; however, such an effort must be conducted with rigorous methodology to ensure the representativeness and reliability of the results [18]. This review aims to analyze the association between short telomere length and IPF incidence. This study conducted a systematic review and meta-analysis to determine the influence of telomere length on IPF occurrence, with the goal of providing new insights into early-stage diagnosis and effective therapeutic strategies [19].

## 2 METHOD

### 2.1 Study Design

A systematic review and meta-analysis were performed according to the guidelines of the Preferred Reporting Items for Systematic Reviews and Meta-Analysis (PRISMA) statement [20].

### 2.2 Search Strategy and Selection Criteria

A systematic online search was conducted on three scientific databases: PubMed, Scopus, and Cochrane, to find observational studies reporting on the association of telomere length with IPF findings. The search was performed on June 9, 2024, with no restriction on publication year. The search was conducted using MeSH terms consisting of several domains: idiopathic pulmonary fibrosis and telomere length.

### 2.3 Inclusion and Exclusion Criteria

#### 2.3.1 Inclusion Criteria

(1) Original articles consisting of telomere length evaluation for idiopathic pulmonary fibrosis.

(2) Study population includes patients with fibrotic ILD diagnosis.

 (3) Telomere length ratio presented.

 (4) Study design, case-control or cohort.

#### 2.3.2 Exclusion Criteria

 (1) No quantitative result of telomere length measurement.

 (2) Study without healthy controls.

(3) Case reports, conference abstracts, and reviews.

For duplicate publication, the study with the largest sample size was included.

### 2.4 Data Extraction

Data extraction was conducted on the included articles that met the inclusion and exclusion criteria. For each article selected, a reviewer extracts information using a standardized form. The following items were extracted for synthesis: title, study design, location, population, number of case and control subjects, measurement method, sample, and telomere length ratio. The second reviewer confirmed the accuracy of the data extractions.

### 2.5 Study Outcome

The primary outcome was the identification of telomere length association with idiopathic pulmonary fibrosis findings. The telomere length outcome was reported in relation to the standard/normal telomere length.

### 2.6 Quality Assessment

Two authors conducted a quality assessment using the Newcastle-Ottawa Scale (NOS) [21]. The instrument is scored by evaluating three domains: selection, comparability, and outcome. Each domain contains four, one, and three questions, respectively. The questions could be graded with one or two stars depending on the domain guidance and quality of the study. The highest quality will be given a maximum of 9 points.

### 2.7 Statistical Analysis

A meta-analysis using a random effects model was conducted for all studies. Heterogeneity among the studies was evaluated using I^2^ statistics, with a p-value < 0.05 deemed significant. I^2^ values of 75% or higher were considered indicative of substantial heterogeneity. Continuous variables and 95% confidence intervals were utilized as summary statistics to assess the mean of telomere length. All analyses were carried out with Review Manager 5.4.1.

## 3 RESULT

### 3.1 Search Results

**Fig. (1):**
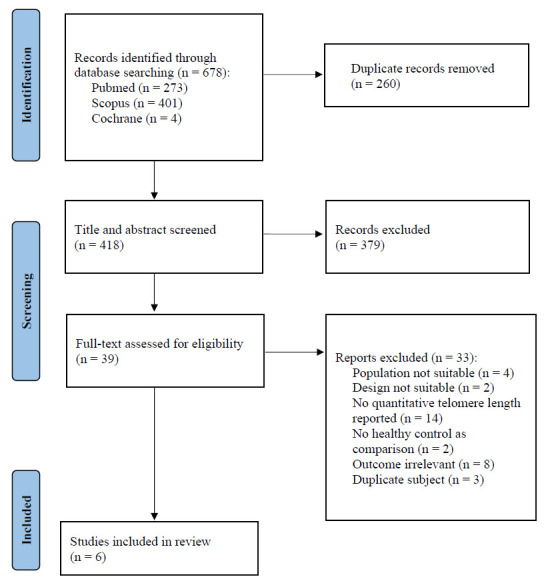
PRISMA flow diagram of study selection process.

The initial search produced 678 potentially relevant articles, and 260 records were retrieved after removing duplicates (Fig. **1**). A total of 379 articles were excluded due to the discordance with the inclusion/exclusion criteria, resulting in 39 eligible articles for full-text screening. After careful evaluation, 33 articles were removed. Eventually, six articles were included in this current systematic review and entered the meta-analysis process.

#### 3.1.1 Study Characteristics

The review consists of four case controls and two cohort studies. These studies were conducted in Asia (*n = 2)*, Europe (*n = 3*), and America (n = 1). All candidates with an IPF diagnosis were included in this review. The telomere length of the majority of patients was identified from peripheral blood leukocytes using qPCR (*n = 3*) and real-time PCR (*n = 1*). The other two studies used lung tissue samples. Results were reported in telomere length ratio relative to reference DNA (T/S). The results were compared with age-matched controls to analyze the difference Table **1**. Quality assessment results show good quality in all of the papers included.

#### 3.1.2 Telomere Length Association with IPF

**Fig. (2):**
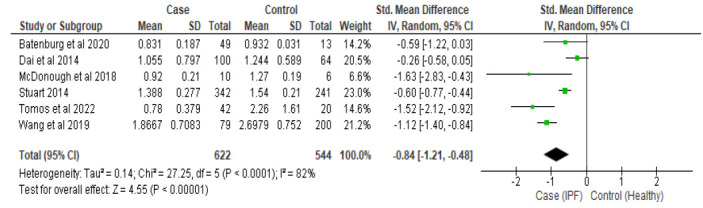
Forest Plot. **Note**: SMD: Standard Mean Deviation; CI: Confidence Interval; SD: Standard Deviation.

Six included studies reported telomere length in a total of 622 IPF patients and 544 healthy controls. Due to significant heterogeneity (*I^2^ =* 82%, *p*<0.00001), a random-effect model was adopted. Pooled analysis showed that IPF patients had significantly shorter telomere length compared to healthy controls (SMD: -0.84, 95%CI -1.21 to -0.48, Z = 4.55, *p<0.00001*) (Fig. **2**).

**Fig. (3):**
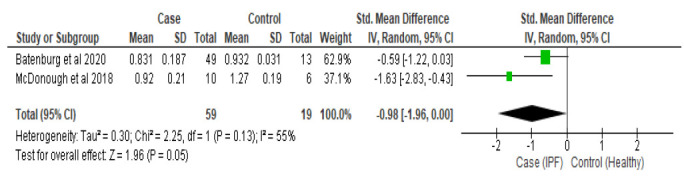
Subgroup analysis of the lung tissue sample. **Note**: SMD: Standard Mean Deviation; CI: Confidence Interval; SD: Standard Deviation.

**Fig. (4):**
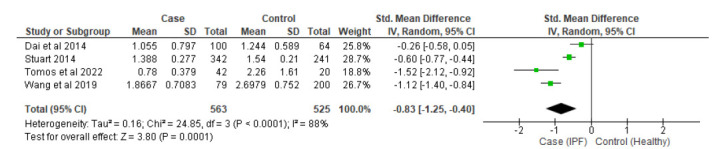
Subgroup analysis of the blood leukocyte sample. **Note**: SMD: Standard Mean Deviation; CI: Confidence Interval; SD: Standard Deviation.

Subgroup analyses were conducted among studies that used lung tissue sample groups (SMD: -0.98, 95%CI -1.96 to -0.00, Z = 1.96, *p = 0.005*) (Fig. **3**) with significantly shorter telomeres compared to healthy controls. Similar results were also shown on subgroup analysis of blood leukocyte sample groups (SMD: -0.83, 95%CI -1.25 to -0.40, Z = 3.80, *p=0.0001*) (Fig. **4**). Between the two groups, lung tissue has shorter telomeres compared to leukocytes.

## 4 DISCUSSION

This study shows significantly shorter telomere length in IPF patients compared to healthy controls (p < 0.00001), observed in both peripheral blood and lung tissue. This meta-analysis showed IPF patients have 0.84x shorter telomere length compared to healthy controls. Previous studies suggest that markedly shortened telomeres, comparable to those in mutation carriers, are associated with worse survival and rapid disease progression [22]. However, thresholds that could guide prognosis or therapeutic decisions remain undefined. Thus, the findings of this meta-analysis support telomere shortening as a risk factor for IPF, while future studies are needed to establish clinically meaningful cutoffs.

Telomeres are repetitive sequences of nucleotides at the ends of chromosomes. These nucleotides are tandem repeats of TTAGGG that shorten after every cell division [25]. Telomeres play a role in degradation prevention and genome integrity protection [28]. The process is known as telomere shortening, which mainly correlates with aging. Shortened telomeres limit the replicative and regenerative capacity of cells, causing cellular senescence that leads to age-related disease development [29].

Telomerase is a reverse transcriptase enzyme that can elongate telomeres by adding TTAGGG repeats to chromosome ends [15]. The process prevents the loss of encoded information in a cell. It is active in embryonic stem cells and mostly silenced after birth [30]. Telomerase, which restores telomere length, consists of two major components: telomerase reverse transcriptase encoded by *TERT* and telomerase RNA encoded by *TERC* [31]. Several other genes, also known as telomere stabilization, such as *DKC1*, *PARN*, and *RTELI* [32]. The continuous shortening of telomeres triggers p53-dependent DNA damage response activation, causing cell senescence or apoptosis that leads to various diseases such as IPF [33, 34].

### 4.1 Telomere Shortening in IPF

IPF is a chronic lung disease characterized by irreversible fibrosis [35]. This occurs due to repetitive injury that causes the bronchoalveolar epithelium to be replaced [36, 37]. Alteration of lung tissue into fibrotic tissue induces lung incapacity to regenerate, leading to idiopathic deterioration and organ failure [23]. Shortened telomeres hinder the healing process, causing fibrotic tissue to persist or even extend [23].

The mechanisms by which telomere defects contribute to lung disease remain unclear. Issues with telomere maintenance have been associated with epithelial cell aging (senescence) and a reduced ability to repair epithelial injuries [32, 38]. With repeated cell divisions, telomeres gradually shorten, ultimately triggering DNA damage pathways that lead to senescence and apoptosis [32, 39]. While cellular senescence can be beneficial in some contexts, premature senescence disrupts the balance of lung epithelial cells, potentially activating lung remodeling processes that result in fibrotic damage [32, 40].

At the cellular level, telomere shortening causes dysfunction in *Alveolar Epithelial Cell Type II* (AEC2s) [19]. Hence, generates spontaneous pulmonary fibrosis through two pathways. First, Cell dysfunction leads to pro-fibrotic responses through senescence-associated secretory phenotype (SASP) [19, 41]. Afterward, it triggers fibrocytes that differentiate into fibroblasts, myofibroblasts, and innate immune cells at the fibrotic lesion site [19]. Second, failure of AEC2s disturbed new alveolar regeneration, leading to increased lung mechanical tension. The arising tension activates the TGF-β signaling loop that increases TGF-β, myofibroblast differentiation, and fibrotic lesions in the lung tissue [19, 42]. This mechanism triggers extracellular matrix filament deposition in lung parenchyma [43]. It is reported that increased total collagen and chromosomal damage lead to elastin deposition and structural disease severity [44].

IPF cases are known to be inherited with an autosomal dominant pattern. *TERT* and *TERC* mutations were identified in 15% IPF families and 2% sporadic cases [39, 45]. However, IPF cases are not only influenced by aging but also by environmental factors. Viral infection, smoking, and occupational exposure could increase IPF risk [46, 47]. The percentage of former or current smokers in IPF cases ranges from 41% to 83%. Exposure to stone, wood, metal, and organic dust also includes IPF risk factors [43].

This study demonstrates a significant correlation between telomere shortening and the occurrence of pulmonary fibrosis. Duckworth *et al*. [48] reported a four-fold increased likelihood of developing IPF in subjects with shortened telomeres *(p=0.0031).* A replication cohort was conducted in up to two thousand IPF subjects, showing a twelve-fold likelihood of IPF in subjects with shortened telomeres. The shortened telomeres were greater compared to this study.

IPF subjects with TERT and TERC variant mutation reported to have shorter telomere length *(p<0.05*) [22]. The length difference is nearly two-fold, although there is no family history of IPF. Jonathan *et al.* [37] also reported shorter alveolar epithelium telomeres in mutated IPF patients (*p= 0.013*). The mutations can appear as genetic carriers or happen sporadically. These mutations disrupt telomerase activity, causing telomeres to shorten. However, short telomeres could also be present even though mutations are not found. The cases were found in 24% familial IPF and 23% sporadic IPF, suggesting other gene mutations in IPF [49].

This study reported steeper telomere shortening in lung tissue compared to peripheral blood. Batenburg *et al.* [50] studied telomere length differences in lung and other tissues among IPF patients. The shortest telomere length was present in lung tissue compared to kidney, thyroid, liver, and bladder. Moreover, telomere shortening was found to be significantly greater in fibrotic lung areas. These findings suggest an association between the fibrotic process and cellular telomere length.

Several studies show poor prognosis in ILD patients, such as IPF and CTD-ILD, that had shortened telomere length [15]. Tesolato *et al.* [51] reported that IPF patients experienced more death events and showed higher mortality rates and poorer survival (*p = 0.464*) were found in telomere patients. Snetselaar *et al.* [52] investigated the association between survival time and ATC2 telomere length. Patients with a shorter alveolar telomere showed decreased median survival of 22 months and lived 41 months shorter than patients with a higher ratio (p=0.003).

### 4.2 Clinical Relevance of Telomere Length

Despite the evolving knowledge of telomere examination in IPF patients, the utility in clinical settings is still controversial. Pulmonary fibrosis probability remains high despite no telomere mutations were found. Goldman *et al.* [53] reported inheritance of telomere length with no mutation findings. The study shows that telomere shortening alone is a heritable trait, which might shorten progressively over generations. However, the disease rarely manifests before the age of 40. Newton *et al.* [54] reported that telomere length could be used as a treatment predictor of immunosuppressive response therapy. Patients with long-term immunosuppressive treatment who have short telomere findings have worse survival compared to those with preserved telomeres. Therefore, telomere measurement could be used for personalized treatment approaches.

Family aggregation as an independent predictive factor for telomere shortening. It was observed that lung interstitial alterations occurred in 25% of first-degree relatives of IPF patients [15]. The risk of death is greater among first-degree relatives compared to second and third [55, 56]. Telomere length in peripheral blood cells suggests a representation of the overall telomere in the individual, including lung tissue.

### 4.3 Limitation

This study has several limitations. The small number of included studies and subjects may affect the generalizability of the findings, particularly given the demographic diversity and variation in disease stages among the study populations. Such variability may have influenced the overall outcomes, making it difficult to establish uniform patterns or recommendations. In addition, the diverse study designs, including both case–control and cohort studies, may introduce bias due to their differing strengths and limitations in establishing causal relationships. Finally, this meta-analysis restricted the criteria to studies reporting quantitative telomere length to allow for pooled analysis; however, this approach may have led to the exclusion of potentially informative studies that could have strengthened the overall conclusions.

Future research with larger, more homogeneous populations is needed to validate these findings and enhance the reliability of the conclusions. In addition, studies should consider incorporating telomere length measurement, particularly from peripheral blood, into risk stratification models for early detection of IPF, as this may help identify high-risk individuals before advanced disease develops [57]. Prospective cohort studies are also recommended to validate telomere length as a predictive biomarker, clarifying whether shortened telomeres are associated with survival outcomes and treatment response, thereby enabling more individualized therapeutic strategies [58].

## CONCLUSION

This study demonstrates that patients with idiopathic pulmonary fibrosis (IPF) have markedly shorter telomere lengths compared to healthy controls, supporting the role of telomere attrition as a potential risk factor for IPF development. These findings highlight the value of telomere assessment not only for early detection but also as a potential predictive biomarker for clinical outcomes. However, the interpretation of these results is limited by the small number of included studies, variability in study designs, and heterogeneity in patient characteristics, which may influence the generalizability of the findings. Future research involving larger, more homogeneous cohorts is needed to validate these associations, establish clinically meaningful telomere length thresholds, and clarify the prognostic value of telomere shortening for clinical outcomes in IPF.

## Figures and Tables

**Table 1:** Study Characteristics

**Study/Refs.**	**Region**	**Design**	**Population**	**Case**		**Control**		**Method**	**Sample**	**NOS**
				**N**	**TL**	**N**	**TL**			
Dai *et al*, 2014 [22]	Asia	Case Control	Sporadic IPF	100	1.055 ± 0.797	64	1.244 ± 0.589	Real-Time PCR	Lymphocyte	9
Tomos *et al*, 2022 [23]	Europe	Case Control	IPF	42	0.78 ± 0.379	20	2.26 ± 1.61	qPCR	Leukocyte	7
Wang *et al*, 2019 [24]	Asia	Cohort	IPF	79	1.867 ± 0.708	200	2.698 ± 0.752	qPCR	Leukocyte	8
McDonough *et al*, 2018 [25]	Europe	Case Control	IPF	10	0.92 ± 0.21	6	1.27 ± 0.19	Real-Time PCR	Lung tissue	8
van Batenburg *et al,* 2021 [26]	Europe	Case Control	IPF TERT-PF	49	0.831 ± 0.187	13	0.932 ± 0.031	MMqPCR	Lung tissue	7
Stuart *et al*, 2014 [27]	America	Cohort	IPF	342	1.388 ± 0.277	241	1.54 ± 0.21	qPCR	Leukocyte	8

## Data Availability

The data and supportive information are available within the article.

## LIST OF ABBREVIATIONS

IPF: Idiopathic Pulmonary Fibrosis
NOS: Newcastle-Ottawa Scale
PRISMA: Preferred Reporting Items for Systematic Reviews and Meta-Analysis

## STANDARDS OF REPORTING

PRISMA guidelines and methodology were followed.

## SUPPLEMENTARY MATERIAL

PRISMA checklist is available as supplementary material on the publisher’s website along with the published article.
